# Mental imagery of object motion in weightlessness

**DOI:** 10.1038/s41526-021-00179-z

**Published:** 2021-12-03

**Authors:** Silvio Gravano, Francesco Lacquaniti, Myrka Zago

**Affiliations:** 1 Laboratory of Neuromotor Physiology, IRCCS Santa Lucia Foundation, 00179 Rome, Italy; 2 Department of Systems Medicine and Center of Space BioMedicine, University of Rome Tor Vergata, 00133 Rome, Italy; 3 Department of Civil Engineering and Computer Science Engineering & Center of Space BioMedicine, University of Rome Tor Vergata, 00133 Rome, Italy

**Keywords:** Physiology, Neuroscience

## Abstract

Mental imagery represents a potential countermeasure for sensorimotor and cognitive dysfunctions due to spaceflight. It might help train people to deal with conditions unique to spaceflight. Thus, dynamic interactions with the inertial motion of weightless objects are only experienced in weightlessness but can be simulated on Earth using mental imagery. Such training might overcome the problem of calibrating fine-grained hand forces and estimating the spatiotemporal parameters of the resulting object motion. Here, a group of astronauts grasped an imaginary ball, threw it against the ceiling or the front wall, and caught it after the bounce, during pre-flight, in-flight, and post-flight experiments. They varied the throwing speed across trials and imagined that the ball moved under Earth’s gravity or weightlessness. We found that the astronauts were able to reproduce qualitative differences between inertial and gravitational motion already on ground, and further adapted their behavior during spaceflight. Thus, they adjusted the throwing speed and the catching time, equivalent to the duration of virtual ball motion, as a function of the imaginary 0 *g* condition versus the imaginary 1 *g* condition. Arm kinematics of the frontal throws further revealed a differential processing of imagined gravity level in terms of the spatial features of the arm and virtual ball trajectories. We suggest that protocols of this kind may facilitate sensorimotor adaptation and help tuning vestibular plasticity in-flight, since mental imagery of gravitational motion is known to engage the vestibular cortex.

## Introduction

Space missions expose humans to the risk of multiple physiological alterations, due to weightlessness, ionizing radiations, confinement, and other stressors^1–4^. In particular, a decline in sensorimotor and cognitive performance can result from the lack of gravity effects^5–7^ and can persist over several weeks after the end of the mission^8^. These alterations are potentially incapacitating during flight and upon landing^9–13^.

Many approaches are followed to develop countermeasures for physiological deconditioning in space (e.g., refs. ^10–13^), including physical exercise^14^, lower-body negative pressure^15^, haptic and proprioceptive stimuli^16^, and artificial gravity^17^. Recently, the use of mental imagery has been also advocated to prepare for the rapid changes in gravitational forces after launch, to reduce the adverse effects of weightlessness exposition, and to prepare for landing^18–20^. Imagery is a voluntary mental experience of objects, scenes, or actions that are not present to our senses. It has proven very useful for sports training and rehabilitation of neurological disorders^21–23^. Motor imagery can enhance physical practice in both terrestrial^23^ and astronaut populations^18,24^.

Mental imagery might be especially useful for training people to deal with conditions that are experienced only in weightlessness. For instance, motor imagery has been used to test the ability of naïve subjects to imagine rotating their body while floating above the floor^25^. The dynamic interaction of different body parts with weightless, free-floating objects is another condition that is not normally experienced under Earth gravity (e.g., neutral buoyancy simulations involve a significant viscous resistance that does not apply to spaceflight), but can be simulated using mental imagery. Astronauts and cosmonauts face the problem of interaction with free-floating objects routinely, as when they shift various tools and equipment on-board. To do so effectively, they must produce lower forces to impart lower momenta to the handled object than those required on Earth, else they risk damaging equipment and/or injuring themselves^26,27^. In general, they must produce finely graded arm movements as part of their duties^28^. However, it is known that people may initially encounter difficulties in calibrating the forces required to shift objects or move around the space vehicle, and they may need some considerable time to adapt their control strategies to weightlessness, as well as to re-adapt upon return to Earth^26,29,30^. Interestingly, some of these difficulties were documented in the hilarious yet accurate animation of *Man in Space* (1955), a television episode resulting from the collaboration between Walt Disney and Wernher von Braun, produced well before any human flew to space. A training program for astronauts has been recommended^30^ to encourage fine-graded motions in order to increase controllability and reduce the risk of injury.

In particular, the control of hand forces poses special problems in weightlessness. During object manipulation in weightlessness, the load force at the fingertips decreases by an amount equal to the weight of the object. Therefore, the minimum grip force required to avoid object slippage from the fingers is lower than on ground. Paradoxically, instead, when first exposed to 0 *g* during parabolic flight, subjects (especially, but not exclusively, the inexperienced ones) adopt the opposite strategy: they increase the grip force relative to 1 *g*, although the temporal coupling between grip and load force is preserved^31–35^. With practice, they adapt by progressively decreasing the grip forces^31,32^. Grip-force adaptation to weightlessness is especially compromised when object manipulation must be coordinated with arm movements involving significant inertial torque loads^33,36,37^. Moreover, persistent alterations are observed when subjects must respond to a target force displayed on a screen by pushing an isometric joystick. In this case, subjects produce significantly higher hand forces at 0 *g* than 1 *g*, and they do not adapt even after several trials^28,38^, possibly because of altered vestibular input^39,40^.

The correct estimate of the time duration of object motion represents another critical issue in weightlessness. People are accustomed through a life-long experience that unsupported objects accelerate downward due to Earth’s gravity. When gravity effects are absent, however, “up” and “down” no longer exist, and objects move at constant speed in the direction of the initial force. According to anecdotal reports, some novice astronauts and cosmonauts expect unconsciously that an object slipping from their hand should fall “down” instead of moving forward inertially^41^. Objective measurement of the behavioral effects of a prior bias of persistent Earth gravity effects was obtained during the Neurolab mission^42^. Astronauts were asked to catch a ball projected from the ceiling towards them at random, constant speeds. They systematically underestimated the motion duration of the incoming ball and activated their arm muscles too early as predicted by an internal model of gravity effects. Premature responses were observed for all tests during the 15 days of the mission, but there was evidence of adaptation towards the end of the mission^42^. In addition, some astronauts and cosmonauts have reported a “time compression syndrome” in orbit, elapsed time being perceived as compressed relative to the corresponding perceptions during training on ground^3^. In parabolic flight, Clément^43^ found that the durations of different stimuli in a duration-reproduction task were significantly underestimated in 0 *g* compared with 1 *g*.

In sum, the combination of inappropriately high forces when manipulating objects with the possible underestimate of the duration of the resulting object motion may be detrimental for in-flight mission operations. However, since mental imagery engages brain regions partially distinct from those involved in motor interaction with real objects^44–46^, we hypothesize that visual imagery might overcome the limitations of perceptual-motor interactions with weightless objects. If so, mental imagery might be useful to train people to deal with weightless objects in preparation for a space mission, as well as during the mission to facilitate space adaptation.

**Fig. 1:**
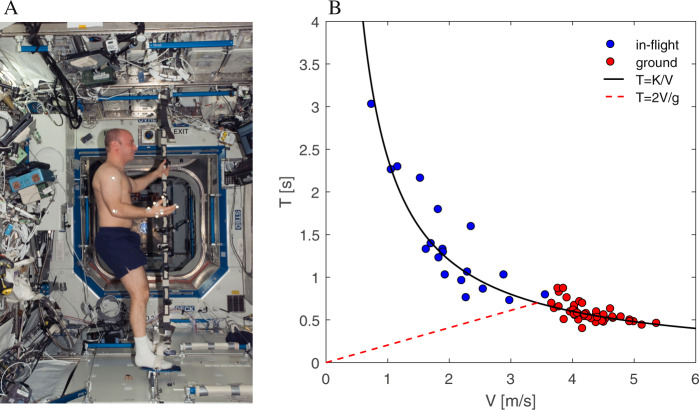
Setup. **A** Picture of astronaut Garrett Reisman on the ISS, performing the protocol of throwing the imaginary ball to the ceiling and catching it on the rebound. Written informed consent for the publication of the picture was obtained from Garrett Reisman. Image courtesy of NASA. **B** Throws of a real ball on ground (red) and in-flight (blue). Black continuous and red dashed curves correspond to *T* = *K/V* and *T* = 2 *V/g*, respectively. *K* = 2.4 m (mean path length of ball trajectory, see text).

With this in mind, we put forth a protocol of mental imagery and pantomime, actual arm actions being performed on virtual objects to mimic the interaction with a ball^47,48^. In the main protocol, the participants are asked to grasp an imaginary ball in the hand, to throw it “up” so as to bounce on the ceiling, and to catch it on the fly when they think it has come back to be grasped (Fig. 1A). They must vary the throwing force and speed across trials and, in different blocks of trials, to imagine that the ball moves under Earth gravity (1 *g*) or weightlessness (0 *g*). An impulsive force exerted by the hand on a ball results in a change of its momentum. The start and end of ball motion are implicitly signaled by the throwing and catching actions, respectively. Accordingly, the relationship between the overall duration (*T*) of ball motion (from throwing to catching) and the throwing speed (*V*) reflects the kinematics of the ball^47^.

At 0 *g* (assuming no air resistance and a constant elastic bounce against the ceiling), *T* is related to *V* by the hyperbolic equation *T* = *K/V*, where *K* corresponds to the distance between the ceiling and the hand at the time of release plus the distance at the time of catch, yielding the total path length of travel of the ball. Thus, the ball can reach the ceiling and bounce back when thrown “up” at any speed, even at very low speeds. At 1 *g*, *T* is related to *V* by a different equation, $T = \frac{2}{g}\left( {V - \sqrt {V^2 - gK} } \right)$. This implies that there is a minimum throwing speed ($V_{{\mathrm{min}}} = \sqrt {gK}$) below which the ball would fall back without contacting the ceiling. For $V \,>\, V_{{\mathrm{min}}}$ and relatively low values of *K* (as in the case of the present experiments), *T* values expected at 1 *g* are very similar to those expected at 0 *g*. This can be appreciated in Fig. 1B plotting exemplary results of throws of a real ball against the ceiling on ground (red dots) and in-flight (blue dots). Despite inter-trial variability of both ball path length and elastic bounce on the ceiling, the data-points for both 1 *g* and 0 *g* conditions are roughly distributed along the hyperbolic function (black curve) with *K* equal to the mean path length. However, as expected, the data obtained at 0 *g* encompass *V* values considerably lower and *T* values considerably longer than the data obtained at 1 *g*.

Of course, in contrast with the results of throwing a real ball, throwing speed *V* and duration of motion *T* of a task involving an imaginary ball are not constrained by physics to covary uniquely: instead, *T* can vary with *V* arbitrarily as a function of the mental model used by each individual. Here, we report the results obtained with the protocol of mental imagery described above in seven astronauts before, during, and after their mission on-board the International Space Station (ISS). Four more astronauts were backups and carried out only the pre-flight experiments. For the sake of comparison, we also report the results of eight control subjects who performed the experiment in our laboratory^47^. One additional astronaut performed a variant of the protocol, throwing the imaginary ball against the front wall instead of the ceiling. This variant differed from the main protocol also in the arm kinematics, the throws being underarm in the main protocol and overarm in the variant.

We hypothesized that, with both protocols, the astronauts were able to simulate qualitatively the characteristics of inertial motion of weightless objects already on ground, and that they further adapted their behavior to the real weightless conditions of spaceflight. We also hypothesized that they could reproduce some of the key differences between target motion under weightlessness and under gravity. In particular, on average they should throw the virtual ball at a lower speed (due to lower impulsive forces of the hand) and should wait for a longer time before catching the ball after the rebound for the imaginary 0 *g* condition than the imaginary 1 *g* condition, as in the case of the throws of a real ball in Fig. 1B. Another prediction concerns the protocol of throwing the imaginary ball against the front wall. Here, the presence or absence of gravity effects constrains the spatial characteristics of the virtual trajectory of the ball. Under Earth gravity, the possible trajectories back and forth from the target would be parabolas. In weightlessness, however, all trajectories should be straight lines. Therefore, adjustments of the throwing and catching gestures to 0 *g* versus 1 *g* conditions should be apparent in arm kinematics, in addition to the *T* versus *V* functions.

## Methods

### Participants

The study was approved by the Institutional Review Boards of the Italian Space Agency (ASI), NASA Johnson Space Center, European Space Agency (ESA), Human Research Multilateral, and Santa Lucia Foundation. The research was in accord with the Code of Ethics of the World Medical Association (Declaration of Helsinki). Twelve astronauts (11 males, 1 female, 46 ± 4 years old, mean ± SD) were enrolled, provided written informed consent, and completed standard pre-flight medical screening with clearance from their flight surgeon before participating in the study. The individual time points for the experiments of the astronauts are given in Table 1. The time schedule of the experiments differed across participants due to operational and mission constraints. Seven astronauts performed pre-flight, in-flight, and post-flight sessions. One astronaut performed pre-flight and in-flight sessions. Four astronauts performed only pre-flight sessions, as backup participants for the main protocol. Eleven astronauts were right-handed and one (S8) left-handed, as assessed by a 13-item questionnaire derived from the Dutch Handedness Questionnaire^49^. S3, S5, S6, S10, S11, and S12 were rookies at the time of experiment performance, while the other astronauts had previous spaceflight experience. The authors affirm that the astronaut Dr. Garrett Reisman provided written informed consent for publication of his image taken while carrying out the experiment on the ISS (Fig. 1A).

For the sake of comparison, we re-analyzed the data of eight control participants from^47^. These included six females, two males (21 ± 2 years), six right-handed, one left-handed, and one ambidextrous. They performed the main protocol in our laboratory under conditions very similar to those of the astronauts on ground.

### Setup

In four astronauts (S1–S4), hand movements were recorded at 127.75 Hz by means of the HPA (Hand Posture Analyzer) system^50^. This system consists of a lycra glove, instrumented with 15 sensors measuring the flexion-extension angles of the fingers plus the adduction-abduction angle of the thumb (0.4° resolution, 1° accuracy), and an inertial system wrapped at the wrist with three linear accelerometers and three orthogonal gyroscopes. In eight astronauts (S5–S12), hand and arm movements were recorded at 200 Hz by means of the ELITE-S2 system, with a 1-mm accuracy after calibration^51,52^. This system consists of four TV cameras monitoring the 3D position of eight retroreflective markers (15 mm in diameter) attached to the skin overlying the shoulder, elbow (humerus lateral epicondyle), wrist (radial styloid process), metacarpophalangeal joint of the thumb, and index finger, the nail of thumb and index fingers (distal phalanges), and hand dorsum on the third metacarpal bone of the dominant arm. In participant S12 (who performed the variant protocol), additional markers were placed on the metacarpophalangeal joint of the little finger, the nail of middle and ring fingers, ear (tragion), forehead (glabella), chin (menton), neck (C7 vertebra), lower trunk (L4 vertebra), superior iliac crest, anterior and posterior superior iliac spines, hip (greater trochanter), knee (femur lateral epicondyle), and ankle (lateral malleolus). In participants S8, S9, S11, we tracked head movements from on-board video recordings (H264-MPEG-4 AVC) by means of Kinovea software (ver. 0.8.15, Joan Charmant). Pre-flight and post-flight tests were performed at NASA JSC or KSC, in a mockup of the ISS Destiny module or in a laboratory with a fake ceiling placed at the same height (2.16 m) as the ceiling of the ISS module. Control participants performed the experiment in our laboratory with a fake ceiling placed at 2.16 m. Their hand and arm movements were recorded at 100 Hz by means of the Optotrak-3020 system (Northern Digital, Waterloo, Ontario, 100-Hz, spatial accuracy better than 0.1 mm) using the same marker placement as for ELITE-S2.

### Experimental procedures

Participants stood in normal erect (on ground) or quasi-erect (in-flight) posture (Fig. 1A). They faced the experimental PC with the instructions placed at about eye-level, at a distance of about 1.5 m. For in-flight tests, the astronauts performed the task with the feet inserted in restraints fixed to the floor of the station. They could hold a fixed pole or a handle with their nondominant hand to stabilize their posture.

In the starting position, the dominant limb was adducted, quasi-“vertical” along the body side, with the hand supinated. The participants were asked to imagine grasping a tennis ball with the dominant hand as if the ball were physically present. In the main experiments, participants (S1–S11) had to throw the imaginary ball against the ceiling with an underarm movement^47^. The “upward” thrust involved flexing the upper arm, forearm, and wrist, and finally opening the grip so as to virtually release the ball from the hand. They waited with the limb in roughly the same posture as when they threw the ball until they thought that the ball had bounced back from the ceiling, and then they closed the grip so as to virtually grasp the incoming ball on the fly. In the experiments of the variant protocol, the participant (S12) was asked to throw the imaginary ball against a bullseye target attached to the front wall at chin height, about 1 m distance, and to catch it back on the rebound. The required throw involved an overarm movement^53^.

In each trial of all experiments, one of three different levels of throwing force (low, medium, and high) was indicated by a bar of corresponding height displayed on the monitor. In different blocks of trials, the participants were asked to imagine that the motion of the ball was either affected by Earth gravity (1 *g* trials) or unaffected by gravity as in the spacelab (0 *g* trials). Experiments with HPA involved two blocks of 12 trials each, one block for the imaginary 1*g*-condition and one block for the imaginary 0*g*-condition. Experiments with ELITE-S2 involved four blocks of 12 trials each, two blocks for the imaginary 1*g*-condition and two blocks for the imaginary 0*g*-condition. 1 *g* and 0 *g* blocks always alternated between each other. The trial duration was 20 s after the presentation of the instruction on the monitor, the instruction specifying imaginary 1 *g* or 0 *g*, plus the level of throwing force (low, medium, or high). During the first 10 s, the participant was asked to look at the instruction and concentrate on the forthcoming action by mentally simulating it. After a GO signal and over the following 10 s, the participant had to execute the throwing and catching sequence that he/she imagined in the previous phase.

Participants did not practice with a real ball in the context of these experiments, except for one participant (S9) who, outside the protocol schedule, decided to execute a few throws of a real rubber ball (size similar to a tennis ball) against the ISS ceiling after two in-flight experiments (flight-day 6 and 85). This participant performed 7 and 12 throws of the real ball the first and the second time, respectively. These results are plotted in Fig. 1B (blue dots). Control experiments with participants throwing a real ball against either the ceiling or front wall in our laboratory have been reported in previous publications^47,53^. Representative results from one control subject are plotted in Fig. 1B (red dots).

### Data analysis

No data were excluded from the analyses. In the sporadic cases when the participant did not respond to the GO signal, reacted too late due to lack of attention or arm kinematics could not be reconstructed due to missing markers, the trial was marked as missing. All analyses (including the statistical ones) were carried out in MATLAB (MathWorks, Natick, MA, USA). Kinematic data were numerically low-pass filtered (bi-directional, zero-phase second-order Butterworth filter, 10-Hz-cutoff) and time-differentiated. We studied the relationships between the throwing speed (*V*) of each trial and the duration of motion of the imaginary ball to and fro (*T*), since these relationships are revealing of the strategy used as a function of the context. For each trial of throws to the ceiling, we computed *V* as the maximum value of the “vertical” component of the velocity of the index tip. *T*_1_ was the time of occurrence of *V* and corresponded to the time of the virtual release of the ball from the hand. *T*_2_ was the time of virtual ball catch and was computed as the time of occurrence of hand closure around the ball, estimated from the minimum value of the rate of change of the distance between the index tip and the metacarpophalangeal joint of the thumb. *T* was the time interval between these two events, *T* = *T*_2_ – *T*_1_. For frontal throws, *V* corresponded to the maximum value of the modulus of the velocity of the ring fingertip, and *T*_2_ corresponded to the time of minimum angular velocity of the upper arm segment in the sagittal plane. These estimates have been previously validated in laboratory experiments involving throws of a real ball against the ceiling or a wall, where we monitored the kinematics of both the hand and a real ball^47,53,54^.

Next, we best-fitted (minimum root-mean-square-error RMSE) the relationship between the *T* and *V* values of all trials of each condition and experiment with the following equation:

1 $$T = \frac{K}{V}$$

Equation 1 describes motion at a constant speed, such as in the absence of gravity effects and air resistance, and assuming an elastic bounce of the ball against the ceiling or the wall with a constant restitution coefficient. For a physically realistic throw, *K* would correspond to the total path length of the ball motion. In our experiments, this corresponds to the distance between the ceiling (or the wall in the variant protocol) and the hand at the time of release plus the same distance at the time of catch. We estimated these distances in the experiments with ELITE-S2 using the measured spatial coordinates of the hand at release and catch times and assuming a nominal contact point of the ball at bounce time located on the vertical line of the throwing position for the main protocol and on the horizontal line for the variant protocol. Notice, however, that these estimates are approximate since the trajectory of the imaginary ball was unknown. Moreover, we could not estimate these distances in the experiments with HPA, because this system does not provide the spatial coordinates of the hand but only its velocity.

Ball motion in the presence of gravity effects theoretically obeys the following equation (again assuming no air resistance and an elastic bounce of the ball on the ceiling):

2 $$T = \frac{2}{g}\left( {V - \sqrt {V^2 - gK} } \right)$$

However, one can notice in Fig. 1B that experimental throws of a real ball on ground can be adequately fit by Eq. 1, also considering the variability of both ball path length and elastic restitution at contact with the ceiling. Moreover, we previously found that Eq. 1 fit the throws of an imaginary ball at 1 *g* adequately for control participants^47^, and here we found that it fit the present results as well. Indeed, the main difference between 1 *g* and 0 *g* is that gravity dictates a minimum throwing speed below which the ball would fall without contacting the ceiling (or wall):

3 $$V_{{\mathrm{min}}} = \sqrt {gK}$$

We quantified the adequacy of Eq. 1 to fit the observed data of each experiment by computing the normalized RMSE of the predicted T* values versus the observed *T* values in all *n* trials of each condition and experiment:

4 $${\mathrm{NRMSE}} = \frac{{\sqrt {{\Sigma}\left( {T^ \ast - T} \right)^2/n} }}{{\overline T }}$$

$\bar T$ is the mean *T* value over *n* trials. To characterize the rate at which participants adapted to spaceflight, the individual *K*-values of in-flight experiments were normalized (*K*_*n*_) to the mean *K* values of the corresponding pre-flight experiments. Changes of *K*_*n*_ of all experiments and participants with flight-day were best-fitted with:

5 $$K_n^\prime = a\;{{{\mathrm{e}}}}^{(b - {\mathrm{FD}})/\tau } + c$$

where $K_n^\prime$ is the best-fitting value of *K*_*n*_, FD is the flight-day, *τ* is the time-constant, *a, b, c* are constants.

In the ELITE-S2 experiments, we also computed the elevation angles relative to the “vertical” in the movement plane (sagittal) for the upper arm, forearm, metacarpus, and fingers. Head pitch in the sagittal plane was estimated as the angle with the “vertical” of the projected segment connecting the markers on ear tragus and glabella in participant S12, and as the corresponding angle of the segment tragus-lateral eye canthus in participants S8, S9, and S11. In participant S12, we also estimated the prono-supination of the forearm from the coordinates of the markers on this segment. For statistical purposes, we considered the difference between the mean value of prono-supination angle over the last 100 ms before and including the throwing time and the 100 ms following and including the catch time. In this participant, we also considered the difference between the mean values of head pitch over the same time intervals.

### Questionnaires of imagery

In the astronauts who performed pre-flight, in-flight, and post-flight sessions, we administered a paper-and-pencil questionnaire to assess the vividness of mental imagery about one week after the return from the mission, at the time of a post-flight experiment. We used the “Vividness of Mental Imagery Questionnaire” (*VMIQ*^55^), including 24 movement imageries from simple (e.g., standing) to complex (e.g., jumping off a high wall). The questionnaire requires imagining one item at a time from two different perspectives: (i) “watching somebody else” (third-person perspective) and (ii) “doing it yourself” (first-person perspective). Vividness had to be rated by the participants on a five-point Likert-type scale: extreme scores of 1 and 5 reflect extremely vivid images and the inability to produce a mental image, respectively.

### Statistics

Descriptive statistics include mean, SD, and uncertainty given by the 95% confidence interval (CI). Statistical differences between conditions were assessed using either three-way ANOVA with imaginary gravity level, session type, and subject as factors on the data separately averaged over each astronaut, or two-way ANOVA with imaginary gravity level and session type on the pooled results of all corresponding trials of all experiments. Bonferroni multiple comparison correction was used for all post hoc tests. Before hypothesis testing, we carried out a rigorous examination of the distribution of model residuals. Statistical significance of all tests was set at *α* = 0.05.

### Reporting Summary

Further information on research design is available in the Nature Research Reporting Summary linked to this article.

## Results

We first report the results of the main protocol involving throws to the ceiling. Overall, there were five missing trials out of 2208 trials (about 0.2%).

### Relationship between target motion duration and throwing speed

Although the instructions specified three discrete levels of throwing force (low, medium, high), all participants varied the throwing speed *V* widely across repetitions of the same reference force. Importantly, however, throwing speed and catching time could differ appreciably as a function of the imagined gravity level and experimental session.

**Fig. 2:**
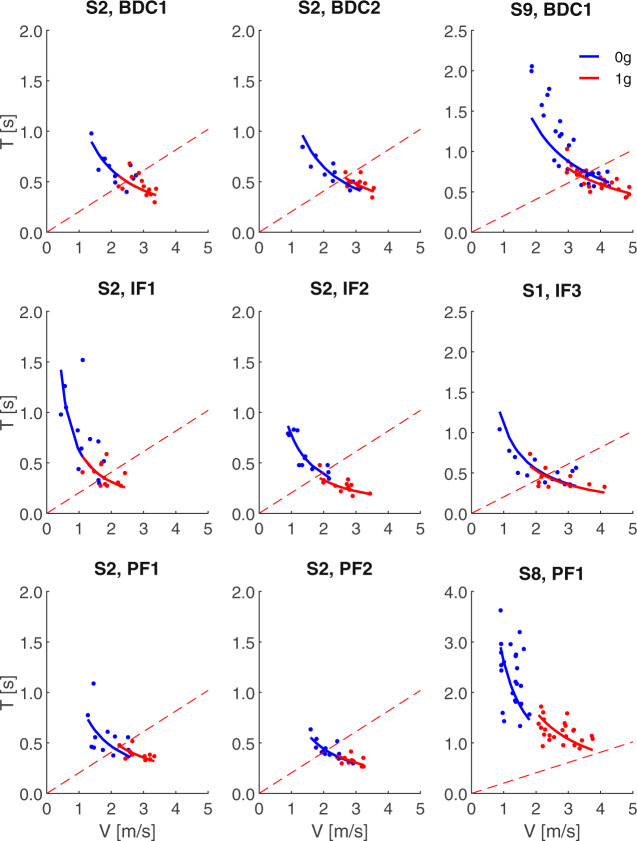
Imaginary motion duration vs. throwing speed. Scatter plots of *T*, *V* values for all trials performed by participants S2, S9, S1, and S8, pre-flight, in-flight, and post-flight, with best-fitting hyperbolic functions of imaginary 0 *g* (blue) and 1 *g* (red) data-points. Dashed curves correspond to *T* = *2* *V/g*.

Figure 2 shows the scatter plots of *T* versus *V*, for all trials of the pre-flight, in-flight, and post-flight experiments performed by one participant (S2), as well as the trials of sample sessions from three other participants (S1, S8, and S9). These plots are qualitatively reminiscent of the *T* versus *V* plots of the throws of a real ball in Fig. 1B, although they can deviate from physics in some relevant respects. We first consider the points of resemblance with the throws of a real ball. The data-points of Fig. 2 are roughly distributed along hyperbolic functions of the form *T* = *K/V* for both the imaginary 0 *g* condition and the imaginary 1 *g* condition. Adherence to the hyperbolic model was better in some sessions than others in the same participant (e.g., IF2 vs. IF1 of S2), or in some participants compared to another one (e.g., S1 vs. S9). Importantly, on average, the participants threw the virtual ball at a lower speed and waited for a longer time before catching back the ball for the imaginary 0 *g* than the imaginary 1 *g* condition, as was the case for the throws of a real ball in Fig. 1B. In several cases, however, the performance was far from being quantitatively consistent with physics. Thus, the minimum throwing speed for the imagined 1 *g* was considerably lower than the theoretical value expected under Earth gravity effects (dashed lines in Fig. 2) in several cases, for instance in S2 IF1 and S8 PF1. Moreover, the parameters *K* of the fitting hyperbolas were congruent with the estimated path length of the imaginary ball only in-flight, as we will see later.

**Fig. 3:**
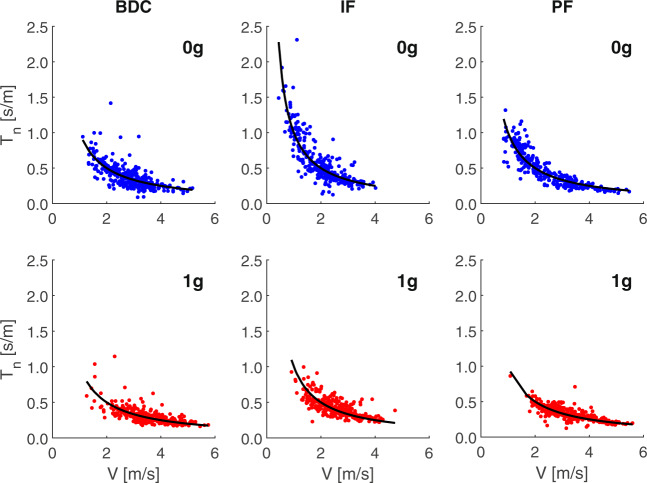
Pooled normalized *T* vs. *V*. Pooled results of all trials of all participants of the main protocol with in-flight experiments, after normalization of the individual *T* values to the best-fitting *K* value of each experiment. Black curves are best-fitting hyperbolic functions over all trials of each condition.

The performance of all participants was qualitatively comparable but differed in the range of individual *V* and *T* values. Therefore, to obtain a global picture (Fig. 3), we pooled the results of all participants with in-flight experiments (*n* = 7, total number of trials = 1824) after normalization of the individual *T* values (see Methods). Despite inter-trial variability, the normalized data-points of the imaginary task are reasonably well-fitted by hyperbolic functions.

We carried out three-way ANOVA with imaginary gravity level, session type, and subject as factors, on the original data separately averaged over each astronaut. We found that *V* differed significantly with imagined gravity level (0 *g*, 1 *g*, *F*(1,11) = 96.22, *P* < 0.001), session (pre-flight, in-flight, post-flight, *F*(2,11) = 164.26, *P* < 0.001), and subject (*F*(6,11) = 55.75, *P* < 0.001). *T* depended significantly on the imagined gravity (*F*(1,11) = 41.92, *P* < 0.001), and subject (*F*(6,11) = 76.09, *P* < 0.001), but did not depend significantly on the session (*F*(2,11) = 0.36, *P* = 0.563).

Since the schedule of the experiments differed across astronauts, we also carried out two-way ANOVA with imaginary gravity level and session type on the pooled results of *V* and *T* (not normalized values) of all corresponding trials of the experiments of participants with in-flight sessions. The results were very consistent with those reported above. *V* differed significantly with both imagined gravity level (*F*(1,1812) = 235.68, *P* < 0.001) and session (*F*(2,1812) = 166.93, *P* < 0.001), with no significant interaction (*F*(2,1812) = 1.51, *P* = 0.222). All post hoc comparisons between imagined gravity levels and between sessions were significant (*P* < 0.001, Bonferroni-corrected). *T* depended significantly on the imagined gravity (*F*(1,1812) = 228.75, *P* < 0.001), but did not depend significantly on the session (*F*(2,1812) = 1.15, *P* = 0.317) or the interaction (*F*(2,1812) = 0.70, *P* = 0.499). All post hoc comparisons between imagined gravity levels were significant (*P* < 0.001, Bonferroni-corrected), but no post hoc comparison between sessions was significant (all *P* > 0.99).

**Fig. 4:**
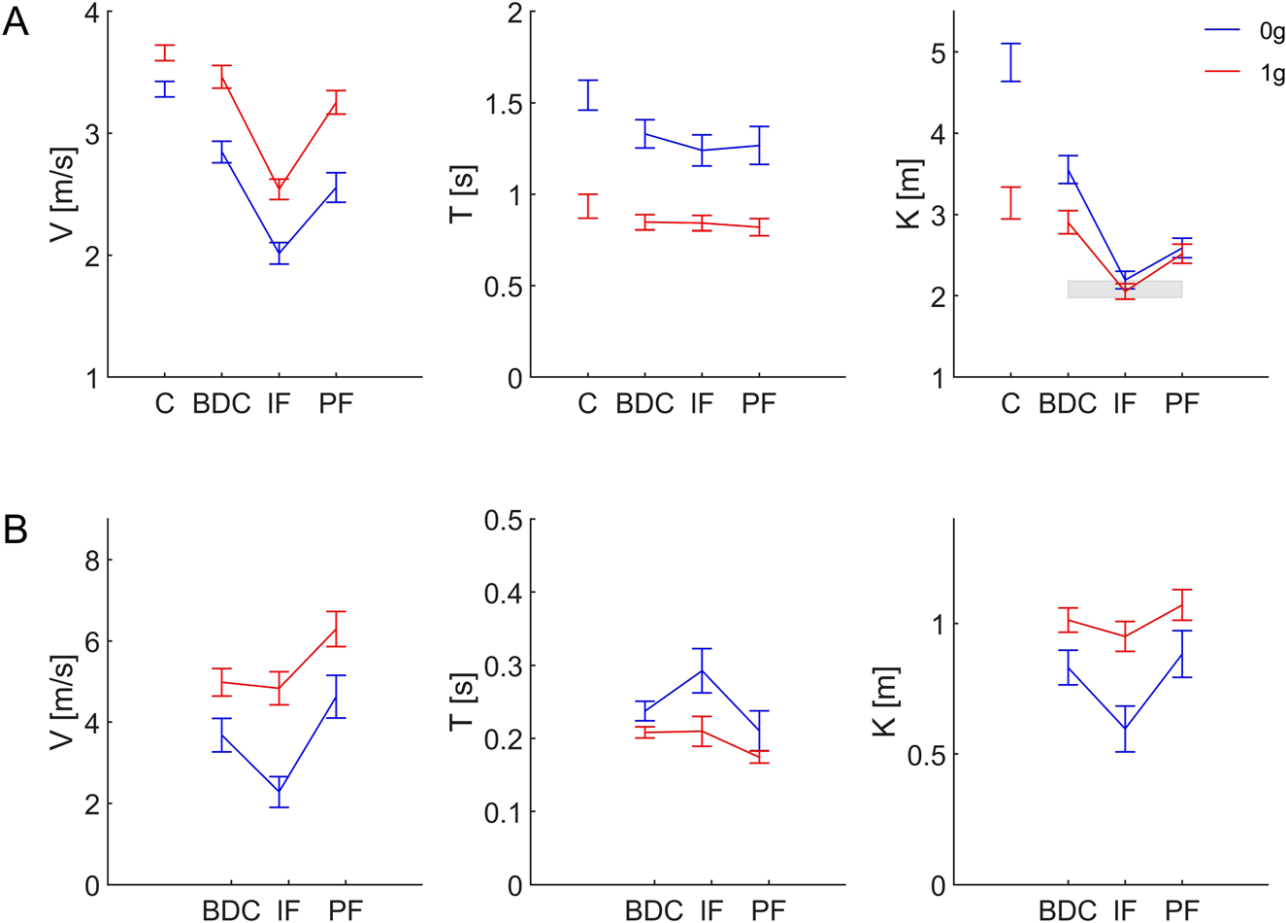
Global performance parameters. Mean values and 95% confidence limits of *V*, *T*, and *K* over all trials, imaginary 0 *g* (blue) and 1 *g* (red), pre-flight, in-flight, and post-flight. **A** Main experiment: seven astronauts and eight controls (labeled C). The gray area denotes the 95% confidence interval for the mean estimated hand-to-ceiling distance. **B** Frontal throws (participant S12).

Figure 4A plots the mean values and 95% confidence limits of *V* and *T* for the different conditions for all trials of the astronauts with in-flight experiments. The results show that, on average, these participants threw the virtual ball at considerably lower speeds and waited for a longer time before catching back the ball when they imagined a 0 *g* condition than a 1 *g* condition. Moreover, during in-flight experiments, they threw the virtual ball at lower speeds than on ground. In the backup astronauts (*n* = 4) who performed the experiment only for baseline data collection on ground, *T* depended significantly on the imagined gravity (*F*(1,382) = 12.51, *P* < 0.001), whereas *V* did not (*F*(1,382) = 1.35, *P* = 0.246).

For the sake of comparison, Fig. 4A also reports the results of control subjects (*n* = 8) who performed the experiment in the laboratory (see Methods). The statistical trend of their responses as a function of imagined gravity level was similar to that exhibited by the astronauts of Fig. 4A. Thus, both *V* and *T* of controls differed significantly with imagined gravity level (*F*(1,380) = 10.63 and 32.09 respectively, *P* < 0.001). However, the mean values of *V* and *T* for the 0 *g* condition in the controls were much higher than the corresponding values of the BDC sessions in the astronauts.

To quantify the extent to which hyperbolic functions *T* = *K/V* fit the scatter plots of *T* versus *V*, we computed the normalized root-mean-square-error NRMSE of the fitting functions. On average, NRMSE was 0.227 (CI[0.206,0.248]), and it did not depend significantly on imagined gravity level (*F*(1,108) = 1.63, *P* = 0.204), session (*F*(2,108) = 2.15, *P* = 0.121), or interaction (*F*(2,108) = 0.51, *P* = 0.599). Post hoc tests did not detect any significant difference of NRMSE between any session comparison (pre-flight, in-flight, and post-flight). However, for the imaginary 0 *g* conditions of all participants, we observed a significant decrease of both the *NRMSE* and the confidence intervals of the regression coefficient between the first and the last BDC, as well as between the first and the last in-flight experiment (by >28% and by >34% for NRMSE and confidence intervals respectively, all *P* < 0.05, paired *t*-test), indicating improved adherence to the hyperbolic functions with practice both pre-flight and in-flight (see for example the results of S2 in Fig. 2). For this analysis, we compared the first and the last experiment of the astronauts who performed at least two experiments in the corresponding session (BDC or in-flight). No such trend was observed for post-flight 0 *g*, nor for any of the imaginary 1 *g* sessions.

The constant *K* of the best-fitting functions differed significantly (two-way ANOVA on the pooled results) with imagined gravity level (*F*(1,1812) = 28.1, *P* < 0.001), session (*F*(2,1812) = 187.11, *P* < 0.001), and interaction (*F*(2,1812) = 15.41, *P* < 0.001, Fig. 4A). The interaction depended on the fact that the imagined gravity level significantly affected only the pre-flight results (*P* < 0.001, post hoc Bonferroni-corrected), but not in-flight and post-flight results (*P* > 0.9). The main changes of *K-*values were related to the session. Thus, *K* was significantly lower in-flight than either pre- or post-flight for both imaginary 0 *g* and 1 *g* (*P* < 0.001, post hoc Bonferroni-corrected). *K-*values post-flight were significantly higher than those in-flight, but lower than at pre-flight (all *P* < 0.001, Fig. 4A). A similar trend was revealed by three-way ANOVA on the data separately averaged over each astronaut. In this case, *K* differed significantly with session (*F*(2,11) = 31.47, *P* < 0.001), and subject (*F*(6,11) = 53.56, *P* < 0.001), but did not differ significantly with imagined gravity level (*F*(1,11) = 0.4, *P* = 0.54) nor was there a significant interaction between session and gravity level (*F*(2,11) = 0.54, *P* = 0.60). In Fig. 4A, it can be noticed that the mean value of *K* for the 0 *g* condition in the controls was considerably higher than the corresponding values in the astronauts.

As remarked in the Introduction, in the case of a ball motion complying with ideal Newtonian mechanics (assuming no air resistance and a constant elastic bounce against the ceiling), *K* corresponds to the total path length of ball motion, back and forth between the hand and the ceiling. We could only estimate path length approximately in the present experiments since the contact point of the imaginary ball with the ceiling was indeterminate. Nevertheless, we computed the 95% confidence interval of an estimated path length in a subset of cases (see Methods), and we plotted it as a theoretical reference in Fig. 4A (gray area). It can be noticed that the mean *K-*values of in-flight experiments for both imaginary 0 *g* and 1 *g* are much closer to the estimated path length than the mean *K-*values of all ground experiments (control subjects, BDC, and post-flight sessions in astronauts). Thus, participants exhibited better compliance of mental imagery of ball motion with the hand-to-ceiling distance during in-flight than ground experiments.

**Fig. 5:**
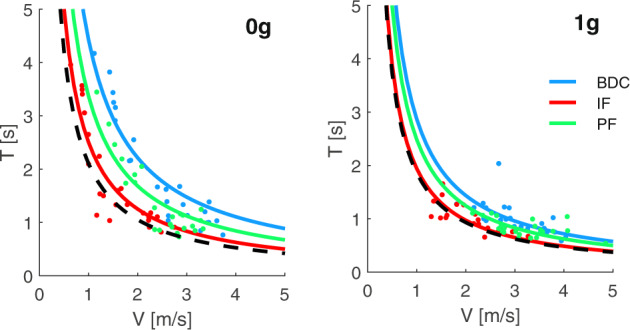
*T* vs. *V* curves pre-flight, in-flight, and post-flight. Results for three different experiments (left to right, imaginary 0 *g* and 1 *g*) performed pre-flight (blue), in-flight (red), and post-flight (green) by participant S11. Notice the shift of *T*-*V* curves in-flight closer to the dashed curves, which depict the predictions based on the hand-to-ceiling distance estimated for this subject in-flight.

The changes of *K-*values in-flight relative to ground have an additional, important implication. The constant *K* of the best-fitting function *T* = *K*/*V* also defines the position of the data in the *T* versus *V* diagram: the smaller is *K*, the closer is the function to the origin and the lower is the throwing speed necessary to obtain a given duration of ball motion. Therefore, lower *K* values in-flight indicate that adjustment to spaceflight involved a shift toward the origin of *T* versus *V* functions interpolating the trials. This shift can be appreciated in Fig. 5 comparing the results for the experiments performed pre-flight, in-flight, and post-flight by one participant. In-flight data-points (red) are closer to the origin than pre-flight data (blue), while post-flight data (green) have an intermediate position, denoting a partial return to the baseline. Thus, *K* values for imagined 0 *g* (1 *g*) data of Fig. 5 were 4.42 (2.87), 2.50 (1.94), and 2.94 (2.4) pre-flight, in-flight, and post-flight, respectively. In Fig. 5, the experimental results are compared with the predictions based on the hand-to-ceiling distance estimated for this subject in-flight (dashed curves). As in the case of the population ensemble of Fig. 4A, in-flight results come closer to the predictions than ground results.

**Fig. 6:**
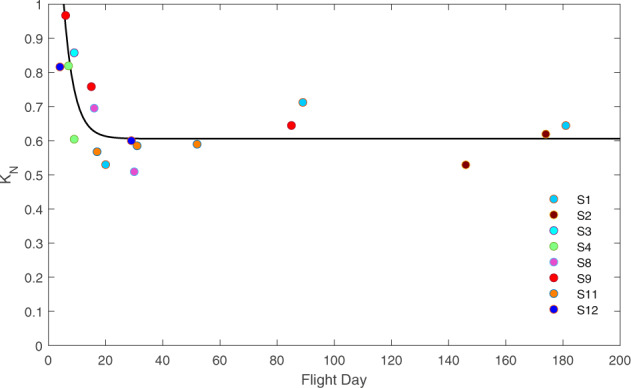
In-flight adaptation. *K*_*N*_, normalized *K-*values of all in-flight experiments plotted as a function of the experimental flight-day. Inset: color-coding of different astronauts. Parameters of exponential fitting (Eq. 5): *τ* = 3.666, *a* = 1.317, *b* = 0.896, *c* = 0.606.

We also searched for trends of changes of *K-*values with days of flight (which are independent of the trends reported above for *NRMSE* and confidence intervals of the regression coefficient). Across participants, adaptation with spaceflight duration was evident for the condition of imaginary 0 *g* ball motion, but not for imaginary 1 *g*. Since the time schedules of the experiments differed across astronauts (see Table 1), we considered the results of all astronauts together under the assumption that they adapted to the flight similarly. Since the individual *K-*values of pre-flight experiments did not show any significant trend with time (*r*^2^ = 0.039, *n* = 16), we normalized the individual *K-*values of in-flight experiments by the mean *K-*values of the corresponding pre-flight experiments. Figure 6 plots the normalized values of *K* for all experiments involving the imaginary 0 *g* condition as a function of the flight-day of the experiment. Participants are denoted by color-coding. It can be noticed that S4, S8, S9 (and S12, see below) showed a substantial decrement of normalized *K* with flight-day, whereas S1, S2, and S11 did not. However, the latter subjects performed all in-flight sessions after the global trend (estimated over all astronauts) has reached a plateau. Indeed, when considered together, the data-points of all participants roughly follow an exponential trend with flight-day (*r*^2^ = 0.622), indicating a decrease of normalized *K-*values by about 40% over the first 17 days, and relatively stable values in the following days (<0.5% change between consecutive days). *K-*values for imaginary 0 *g* at the return from the mission did not exhibit any significant trend with the day after landing (*r*^2^ = 0.020, *n* = 17). No significant trend was found for imaginary 1 *g* ball motion, pre-, in- or post-flight (*r*^2^ < 0.095).

**Table 1:** Session schedule.

Astronaut	BDC	In-flight	Post-flight
S1	−68, −31	20, 89, 181	+8, +14
S2	−155, −86	146, 174	+8, +16
S3	−102	9	+1, +6, +12, +44
S4	−30	7, 9	-
S5	1	-	-
S6	1	-	-
S7	1, 21, 40	-	-
S8	−113, −92, −73, −22	16, 30	+2, +7, +42
S9	−153, −84, −76	6, 15, 85	+2, +6, +67
S10	1, 91, 134	-	-
S11	−147, −56, −13	17, 31, 52	+3, +6, +61
S12	−137, −59	4, 29	+6, +11

Days before launch are indicated for pre-flight experiments (BDC), flight days for in-flight experiments, and days after landing for post-flight experiments. For backup participants, days are relative to the first experiment.

### Arm and head kinematics

**Fig. 7:**
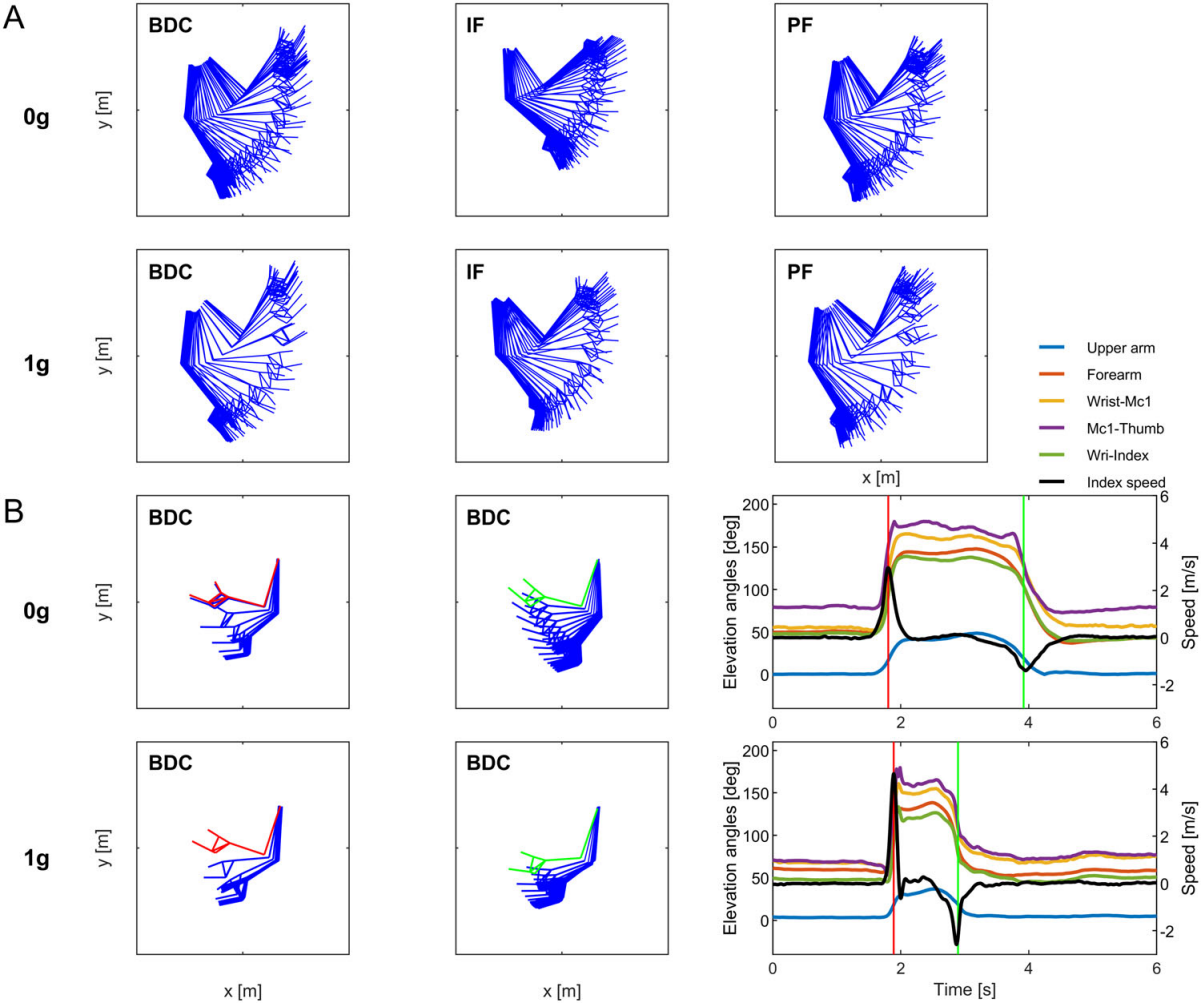
Arm kinematics. **A** Six representative trials of participant S9. Left to right, stick diagrams (every 50 ms) of trials performed pre-flight, in-flight, and post-flight, for imaginary 0 *g* (top) and 1 *g* (bottom). **B** Two representative trials of participant S8 (left-handed). Left: stick diagrams (every 50 ms) prior to throw, and after catch. Red and green sticks correspond to throw and catch times; the other sticks were sampled backward or forward relative to throw and catch, respectively. Right: time course of the elevation angles of the upper arm, forearm, first metacarpus, thumb, index, and “vertical” component of index velocity. Red and green vertical lines correspond to throw and catch times.

In all sessions, throwing the virtual ball in the air was accomplished by relatively stereotyped movements, involving ballistic flexion of the shoulder, elbow, wrist, and metacarpophalangeal joints (Fig. 7A). The time changes of the elevation angles of the limb segments (upper arm, forearm, metacarpus, and fingers) were quasi-synchronous, as were the corresponding changes in the opposite direction at catching time (Fig. 7B). Arm and hand kinematics was qualitatively comparable for the two imaginary gravity levels (0 *g*, 1 *g*), as well as for the experiments performed on ground (pre- and post-flight) and in-flight. In particular, the limb elevation angles covaried tightly in all conditions. However, on average, arm movements tended to be slower for imaginary 0 *g* than 1 *g*, and slower in-flight than on ground, as detailed in the previous section.

**Fig. 8:**
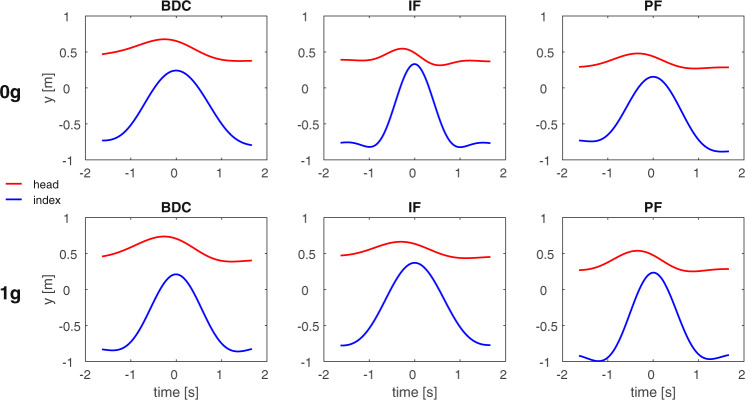
Head and index finger motion. Head and index motion for throwing to the ceiling. Red: time course of the intersection of the ear-eye line with the virtual ball path along the “vertical”. Blue: time course of the “vertical” position of the index finger. Data were averaged over all trials performed pre-flight, in-flight, and post-flight by participant S11.

Arm movements were preceded by head movements. Participants pitched the head up and down by about 60°, as if they tracked part of the imaginary trajectory of the ball. Figure 8 shows exemplary traces (orange) of the time changes of the intersection of the ear-eye line with the virtual ball path (see Methods), compared with the corresponding changes of the “vertical” position of the index finger (blue) for trials performed pre-flight, in-flight and post-flight. In general, the peak velocity of the head movement led to peak velocity of the index. Lead-time depended significantly on the imagined gravity level (*F*(1,240) = 7.72, *P* = 0.0059, two-way ANOVA), but did not depend significantly on session (*F*(2,240) = 0.98, *P* = 0.376) or interaction (*F*(2,240) = 0.89, *P* = 0.412). On average, lead-time was 214 ms (CI[146 ms,282 ms]) and 360 ms (CI[292 ms,428 ms]) for imaginary 0 *g* and 1 *g*, respectively.

### Experiments with frontal throws

The results of the main experiments showed that the participants simulated the qualitative characteristics of inertial motion of weightless objects in the specific case of imaginary motion along the apparent vertical as defined by the ceiling and floor. To extend the findings, we asked one astronaut (S12) to throw the imaginary ball against a target placed on the front wall at shoulder level and to catch it on the rebound. In these experiments, there were five missing trials out of 288 trials (about 1.7%).

**Fig. 9:**
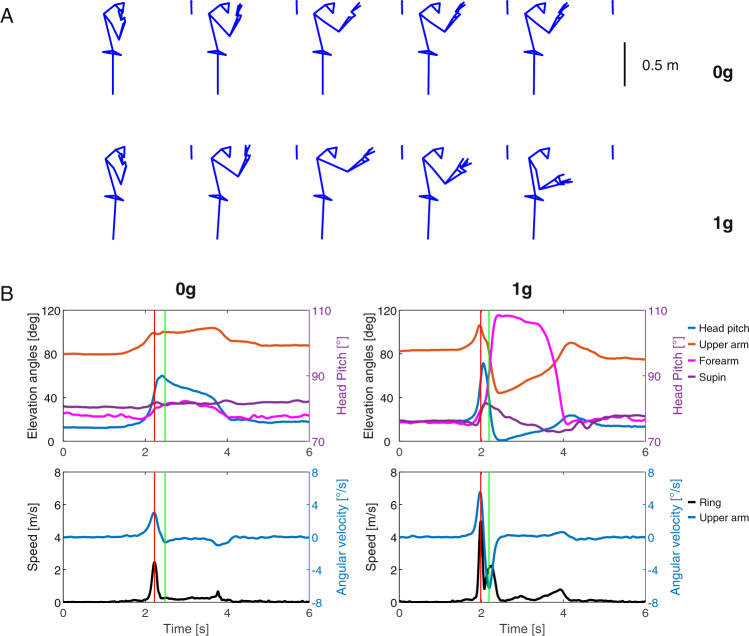
Throws of the imaginary ball to a frontal target. **A** From left to right, stick diagrams at 100 ms before throw, throw, 100 ms after throw, catch, and 100 ms after catch. The short vertical bar on the right of each panel depicts the bullseye target. **B** Upper row: time course of head pitch, upper arm, forearm, and prono-supination angles. Lower row: upper arm angular velocity and ring finger velocity. Red and green vertical lines correspond to throw and catch times. Two representative trials of participant S12, imaginary 0 *g* and 1 *g* condition, pre-flight.

Figure 9A shows the stick diagrams of two representative trials performed pre-flight, and Fig. 9B shows the corresponding changes of head pitch, upper arm, forearm, and prono-supination angles, upper arm angular velocity, and ring finger velocity. The overarm throw involved the forward projection of the hand to impart momentum to the virtual ball. In imaginary 0 *g* trials, the forearm was mid-pronated throughout, from throwing time to catching time. Moreover, the hand caught the virtual ball on the rebound at about the same height from the floor as that at throwing time. On average over all 0 *g* trials (*n* = 140), the difference in “vertical” position of the index finger at throw and catch times was 2.75 cm (CI[2.61 cm,2.89 cm]). In these trials, the head rotated to a very limited extent. Overall, head, arm, and hand gestures were compatible with throwing and catching a ball that moved along a straight path back and forth from the wall, consistent with inertial motion in the absence of gravity effects.

In contrast, in imaginary 1 *g* trials (*n* = 143 trials), the forearm was mid-pronated at throwing time but became mid-supinated at catching time, as in an underarm catch. Moreover, to catch the virtual ball on the rebound, on average the hand was lowered by 17.38 cm (CI[16.64 cm,18.13 cm]). In 1 *g* trials, the head pitched slightly downward after the throw, as if it tracked the imaginary downward trajectory of the ball. Overall, head and arm gestures were qualitatively compatible with throwing and catching a ball that fell downward, consistent with gravity effects.

To assess the statistical differences of the throwing-catching sequence between conditions, we considered the changes of both prono-supination and head pitch between throw and catch (see Methods). We found that prono-supination depended significantly on imagined gravity level (*F*1,278) = 280.79, *P* < 0.001), session (*F*(2,278) = 25.76, *P* < 0.001), and interaction (*F*(2,278) = 29.08, *P* < 0.001). On average, the forearm rotated toward supination by 91.9° (CI[84.7°,99.2°]) in imaginary 1 *g* trials, while it rotated by 20.4° (CI[9.3°,27.4°] in imaginary 0 *g* trials. Notice that prono-supination per se does not fully capture hand orientation in 3D space. Indeed, small amounts of adduction/abduction and axial rotation at the glenohumeral joint, plus flexion-extension, and abduction-adduction at the wrist also contribute to hand orientation^56^.

Head pitch also depended significantly on imagined gravity level (*F*(1,278) = 57.07, *P* < 0.001) and interaction (*F*(2,278) = 4.75, *P* < 0.01), but did not depend significantly on session (*F*(2,278) = 1.17, *P* = 0.31). The head was pitched downward by 5.57° (CI[4.90°,6.25°]) in imaginary 1 *g* trials, while the pitch rotation was 2.59° (CI[2.18°,3.00°]) in imaginary 0 *g* trials. Since the imaginary ball in 1 *g* trials shifted along the vertical by a small extent between the throw and the catch, the corresponding head movements were also of small amplitude compared with those of the main experiment, when the imaginary ball shifted along a considerable vertical path.

The trends of *T*, *V*, and *K-*values were similar to those described for the main experiments, consistent with a differential processing of imaginary gravity level and with adaptation to flight (Fig. 4B). All three parameters differed significantly with imagined gravity level (*F*(1,278) = 116.27, 38.63, and 74.14 for *V*, *T*, and *K* respectively, *P* < 0.001), session (*F*(2,278) = 41.69, 18.08, and 18.66, *P* < 0.001), and interaction (*F*(2,278) = 4.66, 4.42, and 4.0, *P* < 0.02). For in-flight imaginary 0 *g* trials, *V* was significantly lower and *T* significantly higher than the corresponding values at pre- and post-flight (both *P* < 0.001, post hoc Bonferroni-corrected). *V* and *T* of imaginary 1 *g* trials depended less on the session than the corresponding values of 0 *g* trials. *V* in-flight was not significantly different from pre-flight (*P* = 1) but was significantly lower than post-flight (*P* < 0.001, post hoc Bonferroni-corrected). *T* in-flight was not significantly different from either pre-flight (*P* = 1) or post-flight (*P* = 0.152). Also, this participant showed adaptation of *K-*values of 0 *g* trials in-flight, with a decrement over the two successive in-flight experiments. The corresponding data-points (blue in Fig. 6) are roughly aligned with the exponential function fitting the results of the main experiment.

### Rating of imagery vividness

All tested participants reported good levels of the vividness of mental imagery in the questionnaire provided after the experiments. Thus, the mean scores of *VMIQ* were 1.57 (CI[1.40,1.74]) and 1.87 (CI[1.73,2.02]) for the imagery items in the first-person and third-person perspective, respectively, corresponding to scores intermediate between “Perfectly clear and as vivid as normal vision” (score = 1) and “Clear and reasonably vivid” (score = 2) of the scale.

## Discussion

The results are consistent with the hypothesis that the participants were able to simulate mentally the qualitative characteristics of inertial motion of weightless objects already on ground, and that they further adapted their behavior to the real weightless conditions of spaceflight. On average, the participants threw the virtual ball at lower speeds *V* and waited for a longer time *T* before catching back the ball for the imaginary 0 *g* condition than the 1 *g* condition. Moreover, the *T* versus *V* function interpolating the imaginary 0 *g* trials performed during spaceflight shifted closer to the origin compared to the corresponding function of the baseline trials performed on ground. We quantified the shift in terms of the constant *K* of the hyperbolic function fitting *T* versus *V* data-points. Mean *K-*values were significantly lower in-flight than either pre- or post-flight, indicating that the astronauts used a lower throwing speed to obtain a given duration of ball motion. Moreover, the normalized *K-*values of 0 *g* trials pooled across participants decreased exponentially with flight-day, suggesting adaptation with increasing exposure to spaceflight. Practice with the imaginary 0 *g* condition led to improved adherence to the ideal model of inertial motion both on ground and in-flight, as shown by the reduced fitting errors. Finally, the analysis of arm kinematics of the frontal throws revealed a differential processing of imagined gravity level (0 *g* versus 1 *g*) also in terms of the spatial features of the virtual ball trajectory and arm movements.

Agreement of mental imagery with physics was generally qualitative rather than quantitative, and there were cases of significant departure from Newtonian mechanics. Thus, throwing speeds for imagined 1 *g* were often lower than the minimum speed theoretically compatible with throws hitting the ceiling. Also, participants exhibited reasonably accurate compliance of mental imagery with the hand-to-ceiling distance only during in-flight experiments. In the following, we consider the implications of the results, including alternative explanations.

Although the astronauts were instructed to imagine the motion of the virtual ball, they could have followed a simpler strategy of mental calculation^57^ that would result in an inverse relationship between throwing speed and duration of flight of the virtual ball: for a higher throwing speed, wait a longer time before catching. However, there are important clues that the participants did simulate the motion of a virtual ball. First, the data-points of each condition were reasonably well-fitted by a hyperbolic function with a single *K* constant, indicating adherence to a specific mental model of target motion. To replicate these findings, the mental calculation would need to be rather accurate. Furthermore, we observed that the participants of the main experiment pitched the head “up” and “down” by about 60° as if they tracked part of the imaginary trajectory of the ball. Head movements systematically led to arm movements, lead-time being much longer for imaginary 1 *g* than 0 *g* motions, indicating a differential processing of the imaginary gravity level. Similarly, in the experiments with frontal throws, head movements, arm prono-supination and hand position at catch time were all indicative of a differential processing of the imaginary gravity level, rather than of a simple mental calculation of elapsed time.

Since the instructions about the throwing force to apply in each given trial were identical for the 0 *g* and 1 *g* trials, it was the participants’ decision to tune their forces to the imaginary gravity level, resulting in lower throwing speeds for 0 *g* trials, on average. In addition, the mean *V* values for both 0 *g* and 1 *g* trials performed in-flight were lower than the corresponding values for the trials on ground. In line of principle, the latter finding might be related to a general slowing of movements in weightlessness. Previous reports in the literature on this subject are conflicting. Some studies reported a tendency for movement slowing for both vertical and horizontal pointing movements performed in space^58–60^ or parabolic flight^36,61,62^. In particular, Mechtcheriakov et al.^59^ reported significant slowing of aiming arm movements over the initial in-flight sessions and a tendency to have movement durations comparable to pre-flight sessions in later sessions (about 50 days after launch). In other studies, instead, the peak velocity of arm movements was not significantly affected by weightlessness^63–67^. In the Neurolab experiments, Bock et al.^68^ found slowing of pointing movements but not of tracking movements. Overall, it appears that the effects of weightlessness on arm kinematics are task-dependent.

Importantly, a generic slowing of movements cannot account for the shift of the *T*-*V* scatter plots closer to the origin (Fig. 5), resulting in lower values of the *K* parameter for in-flight data (Fig. 4). Had the participants used lower throwing speeds while keeping the same mental image of the virtual ball kinematics in-flight as on ground, the *T*-*V* data-points should have shifted along the same or a similar hyperbolic function in-flight as that found on ground, yielding similar *K-*values.

In theory, mental imagery of ball kinematics might change in relation to a misperception of velocity, time or distance. Thus, simple arithmetic shows that lower values of *K-*values in-flight might depend on either an overestimate of the motion duration of the virtual ball for a given throwing speed or an overestimate of throwing speed for a given ball motion duration. For a given *V*, a hypothetical duration overestimate would result in lower values of *T* than anticipated by the subject. However, if this were the case, we would expect shorter *T* values in-flight than on ground, which was not the case. Moreover, previous studies reported underestimates rather than overestimates of time durations in weightlessness, suggesting a time-compression effect for spaceflight^42,43,69^.

As for hypothetical speed overestimates, these would result in lower values of *V* for a given *T* than anticipated by the subject. Overestimates of movement speed are theoretically possible due to gain changes of sensory inflow from muscle spindles induced by weightlessness. Spindles gain attenuation -due to both reduced otolith influences and mechanical unloading of the body- has been advocated to explain possible alterations of arm and leg motor control, position sense, perceptual judgments of object length, heaviness, and mass^5^. However, the observation that *V*- and *K-*values remained significantly lower than the pre-flight baseline values even several weeks after return to the ground indicates that the adaptation of mental imagery was maintained independently of weightless conditions, indicating a minor role of spindles gain attenuation.

Still another hypothetical explanation for the decrease of *K-*values in-flight is that the participants underestimated the hand-to-ceiling distance when they threw and caught the imaginary ball. Indeed, for a physically realistic throw, the constant *K* corresponds to this distance. However, misperceptions of distances have been reported in the opposite direction during parabolic flight, with overestimates of upward distances at all tested gravity levels, including 0 *g*^70^. Here, the average *K-*values of in-flight experiments did not differ significantly from the estimated summed hand-to-ceiling distances at the throw and catch time, implying that participants implicitly used a correct estimate of these distances in-flight. If anything, the observation that the average *K-*values of ground experiments were higher than the hand-to-ceiling distance would be consistent with distance overestimates on ground^70^.

We remark that, if throwing speed, time duration, or ceiling distance were misrepresented, the parameter *K*, which depends on these perceptual variables, should covary tightly in imaginary 0*g* and 1 *g* trials. In particular, one would expect a similar exponential decay of normalized *K* values with flight-day for both imaginary conditions, which was not the case (see next section). Thus, although misperceptions of the above variables are theoretically possible, a more plausible interpretation is that of a genuine adaptation of mental imagery to spaceflight. Specifically, the astronauts appeared to achieve a better representation of 0 *g* inertial motion in weightlessness and better discrimination relative to 1 *g* gravitational motion.

The results indicate that the participants managed to adjust to spaceflight by shifting the *T* versus *V* function interpolating the imaginary throws closer to the origin for both 0 *g* and 1 *g* trials (Fig. 5). Moreover, considering the overall trend over all astronauts, we found evidence for a further adaptation of normalized *K*-values of 0 *g* trials (but not 1 *g* trials) with prolonged exposure to weightlessness (Fig. 6). This adaptation implied that astronauts employed lower throwing speeds to obtain a given duration of imaginary ball motion, precisely the kind of adaptation advocated in the Introduction as necessary to impart lower momenta to handled objects during spaceflight than on Earth^26,71^.

On the other hand, the results also indicated that mental imagery of target motion under gravity often deviated substantially from physical predictions. Thus, the minimum throwing speed for an imagined 1 *g* was often considerably lower than the theoretical value predicted by Newtonian mechanics. In this respect, the current results are consistent with previous findings in control participants on ground^47,57^ showing that mental imagery of gravity-accelerated motion does not conform to the quantitative parameters of Newtonian mechanics. Notice, however, that we cannot expect that the results of mental imagery of 1 *g* replicate all features of Newtonian mechanics. Indeed, the *T* versus *V* function of a real ball motion under any gravity value (not just 1 *g*) is non-monotonic, so that *T* increases linearly with *V* without contacting the ceiling up to a threshold speed (see dashed lines in Fig. 1B and Fig. 2), while *T* decreases non-linearly with increasing *V* above that threshold. However, we instructed our participants to throw the imaginary ball in such a manner as to contact the ceiling, so that we would expect that *T* should decrease with increasing *V* for imagined 1 *g* just as for imagined 0 *g*, which is what we found.

On Earth, the vertical direction is estimated by a combination of gravity, body, and vision cues, each weighted based on its reliability^72^. In weightlessness, the motor and perceptual “vertical” are often dominated by body-centered cues^73–75^. In the present experiments, there were no gravity-related cues nor visual cues about the imaginary ball trajectory. However, the ISS modules are endowed with abundant visual cues about “up” and “down”, providing a visual reference for the “vertical”. Body-centered and visual cues about the “up” direction were aligned in our experiments so that we cannot distinguish between the relative roles of different references frames for mental imagery.

The *T*-*V* characteristics of the protocol involving frontal throws were qualitatively similar to those of the main protocol, showing that mental imagery of gravitational or inertial motion can occur also for motion directions roughly orthogonal to the visual and body-centered “vertical”. Moreover, the analysis of arm kinematics revealed that the participant threw and caught the imaginary ball as if they anticipated a linear trajectory of the virtual ball for imaginary 0 *g* trials but a downward parabolic trajectory for imaginary 1 *g* trials. Therefore, the presence or absence of gravity effects appears to constrain not only the temporal but also the spatial characteristics of the imaginary trajectory.

The study had some limitations. We reconstructed head movements in the sagittal plane, but we could not monitor eye movements. The time course of adaptation with flight-day should be interpreted cautiously since its analysis relied on between-subjects comparisons, time-schedules being different across participants. We were able to enroll only one astronaut for the frontal throws. We could not discriminate possible differences in performance between rookies (first-time fliers) and experienced astronauts, given the limited number of participants. Three out of four of the backup astronauts were rookies at the time of experiments, but so were three out of seven of the astronauts who performed in-flight experiments (see Methods). Therefore, we do not know whether the different performance of backups relative to the baseline performance (BDC) of astronauts who later performed the main protocol in-flight depended on the lack of previous flight exposure.

An important comparison is that of the astronauts’ performance with the performance of our control subjects (obviously without any spaceflight experience) who carried out the same protocol of throwing an imaginary ball to the ceiling. The control participants showed a statistical trend of the responses as a function of imagined gravity similar to that of the astronauts. However, on average, they employed throwing speeds (and *K*-values) for the 0 *g* condition much higher than those of the astronauts. This difference further highlights the specific fitness of the astronauts to weightlessness, possibly derived from their prior training in parabolic flight or previous experience with spaceflight.

In conclusion, the protocol of mental imagery of inertial motion of weightless objects lends itself to training people in preparation for a space mission, as well as during the mission to facilitate space adaptation. Similarly, mental imagery of gravitational motion might be used during long-term missions to facilitate re-adaptation to gravity effects in preparation for landing. Since the motor cortex shows plastic changes during motor learning by mental training^76^, it is possible that training astronauts with our mental imagery protocols may enhance motor plasticity, progressively inducing adaptation of the hand forces employed to shift objects as a function of the gravity level. Moreover, this or similar protocols of mental imagery may help tuning vestibular plasticity in-flight. The reasoning is as follows. Vestibular sensory deprivation and altered central interpretation of vestibular inputs are key factors underlying several sensorimotor and cognitive impairments during and after spaceflight^5,6^. Recent neuroimaging studies found evidence for vestibular cortex reorganization following spaceflight, suggesting that space-related factors deeply influence the neural correlates of vestibular processing in several brain regions including the insulae^71,77,78^. Moreover, functional magnetic resonance studies showed that the visual effects of gravitational motion are processed in the vestibular cortex, whose hub regions are the insula, retro-insula, and parietal operculum^79,80^. Finally, the imaginary performance of actions normally affected by gravity activates insular regions close to those encoding the visual effects of real gravity^45^. Therefore, the ability to engage the vestibular cortex with mental imagery raises the interesting possibility of modulating central vestibular networks and reducing maladaptive responses to spaceflight. The imagery might modulate vestibular networks using top-down predictive mechanisms of target motion under normal or reduced gravity effects^81^.

## Supplementary information

Reporting Summary

## Data Availability

Data of Fig. 3 including the results of all trials of all deidentified participants of the main protocol with in-flight experiments are deposited at https://figshare.com/s/7e9060139c5a93cdf585.

## Code availability

Custom codes for analyses will be made available upon reasonable request to the corresponding author.
