# Supplementary material for: Mental imagery of object motion in weightlessness
Source: NPJ Microgravity. 2021 Dec 3;7:50. doi: 10.1038/s41526-021-00179-z (PMC8642442; doi:10.1038/s41526-021-00179-z)
Supplement: Supplementary file 1 — Reporting Summary [file 41526_2021_179_MOESM1_ESM.pdf]

nature portfolio

Corresponding author(s): Myrka Zago

Last updated by author(s): Oct 20, 2021

Reporting Summary

Nature Portfolio wishes to improve the reproducibility of the work that we publish. This form provides structure for consistency and transparency in reporting. For further information on Nature Portfolio policies, see our Editorial Policies and the Editorial Policy Checklist.

Statistics

For all statistical analyses, confirm that the following items are present in the figure legend, table legend, main text, or Methods section.

n/a	Confirmed	
☐	☒	The exact sample size (*n*) for each experimental group/condition, given as a discrete number and unit of measurement
☐	☒	A statement on whether measurements were taken from distinct samples or whether the same sample was measured repeatedly
☐	☒	The statistical test(s) used AND whether they are one- or two-sided<br>*Only common tests should be described solely by name; describe more complex techniques in the Methods section.*
☐	☒	A description of all covariates tested
☐	☒	A description of any assumptions or corrections, such as tests of normality and adjustment for multiple comparisons
☐	☒	A full description of the statistical parameters including central tendency (e.g. means) or other basic estimates (e.g. regression coefficient) AND variation (e.g. standard deviation) or associated estimates of uncertainty (e.g. confidence intervals)
☐	☒	For null hypothesis testing, the test statistic (e.g. *F*, *t*, *r*) with confidence intervals, effect sizes, degrees of freedom and *P* value noted<br>*Give P values as exact values whenever suitable.*
☒	☐	For Bayesian analysis, information on the choice of priors and Markov chain Monte Carlo settings
☐	☐	For hierarchical and complex designs, identification of the appropriate level for tests and full reporting of outcomes
☐	☐	Estimates of effect sizes (e.g. Cohen's *d*, Pearson's *r*), indicating how they were calculated

Our web collection on statistics for biologists contains articles on many of the points above.

Software and code

Policy information about availability of computer code

Data collection	In 4 astronauts hand movements were recorded at 127.75 Hz by means of the HPA (Hand Posture Analyzer) system and code(Baldacci et al. 2001). In 8 astronauts, hand and arm movements were recorded at 200 Hz by means of the ELITE-S2 system and code, with a 1-mm accuracy after calibration (Ferrigno et al. 2004; Neri et al. 2014). In three astronauts we tracked head movements from on-board video recordings (H264-MPEG-4 AVC) by means of Kinovea software (ver. 0.8.15, Joan Charmant). In eight control hand and arm movements were recorded at 100 Hz by means of the Optotrak-3020 system (Northern Digital, Waterloo, Ontario, 100-Hz, spatial accuracy better than 0.1 mm).
Data analysis	Matlab (MathWorks, Natick, MA, USA)

For manuscripts utilizing custom algorithms or software that are central to the research but not yet described in published literature, software must be made available to editors and reviewers. We strongly encourage code deposition in a community repository (e.g. GitHub). See the Nature Portfolio guidelines for submitting code & software for further information.

Data

Policy information about availability of data

All manuscripts must include a data availability statement. This statement should provide the following information, where applicable:

- Accession codes, unique identifiers, or web links for publicly available datasets
- A description of any restrictions on data availability
- For clinical datasets or third party data, please ensure that the statement adheres to our policy

Data of Figure 3 including the results of all trials of all participants of the main protocol with in-flight experiments are deposited at https://figshare.com/s/7e9060139c5a93cdf585. The other data will be made available upon reasonable request to the corresponding author.

Field-specific reporting

Please select the one below that is the best fit for your research. If you are not sure, read the appropriate sections before making your selection.

☒ Life sciences ☐ Behavioural & social sciences ☐ Ecological, evolutionary & environmental sciences

For a reference copy of the document with all sections, see nature.com/documents/nr-reporting-summary-flat.pdf

Life sciences study design

All studies must disclose on these points even when the disclosure is negative.

Sample size	Twelve astronauts (11 males, 1 female, 46 ± 4 years old, mean ± SD). Sample size derived from prior studies on astronauts
Data exclusions	No data exclusion. Overall, there were 5 missing trials out of 2208 trials (about 0.2%).
Replication	non astronauts participants
Randomization	NASA scheduled specific sessions
Blinding	Astronaut schedule is public. We deidentified data for further analysis. Independent analysis has been conducted by different person and led to the same results.

Reporting for specific materials, systems and methods

We require information from authors about some types of materials, experimental systems and methods used in many studies. Here, indicate whether each material, system or method listed is relevant to your study. If you are not sure if a list item applies to your research, read the appropriate section before selecting a response.

Materials & experimental systems

n/a	Involved in the study	
☒	☐	Antibodies
☒	☐	Eukaryotic cell lines
☒	☐	Palaeontology and archaeology
☒	☐	Animals and other organisms
☒	☐	Human research participants
☒	☐	Clinical data
☒	☐	Dual use research of concern

Methods

n/a	Involved in the study	
☒	☐	ChIP-seq
☒	☐	Flow cytometry
☒	☐	MRI-based neuroimaging
